# Keeping in touch with the membrane; protein- and lipid-mediated confinement of caveolae to the cell surface

**DOI:** 10.1042/BST20190386

**Published:** 2020-02-12

**Authors:** Madlen Hubert, Elin Larsson, Richard Lundmark

**Affiliations:** Department of Integrative Medical Biology, Umeå University, Umeå SE-901 87, Sweden

**Keywords:** caveolae, caveolin, cholesterol, dynamics, EHD2, pacsin2

## Abstract

Caveolae are small Ω-shaped invaginations of the plasma membrane that play important roles in mechanosensing, lipid homeostasis and signaling. Their typical morphology is characterized by a membrane funnel connecting a spherical bulb to the membrane. Membrane funnels (commonly known as necks and pores) are frequently observed as transient states during fusion and fission of membrane vesicles in cells. However, caveolae display atypical dynamics where the membrane funnel can be stabilized over an extended period of time, resulting in cell surface constrained caveolae. In addition, caveolae are also known to undergo flattening as well as short-range cycles of fission and fusion with the membrane, requiring that the membrane funnel closes or opens up, respectively. This mini-review considers the transition between these different states and highlights the role of the protein and lipid components that have been identified to control the balance between surface association and release of caveolae.

## Introduction

**Figure 1.**
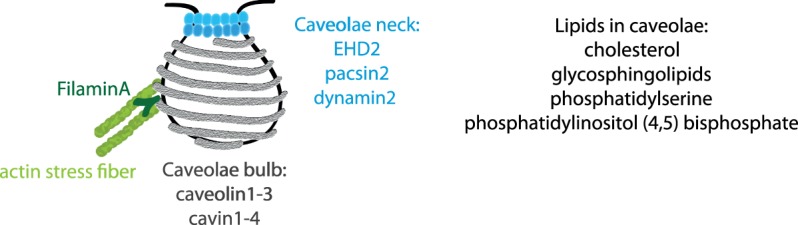
Schematic illustration of caveolae and the associated proteins and lipids discussed in this review.

Caveolae are characteristic small (50–80 nm) invaginations of the plasma membrane enriched in cholesterol, sphingolipids, the integral membrane proteins caveolin1–3 (CAV1–3) and peripherally attached proteins such as cavins (cavin1–4), EH-domain containing protein 2 (EHD2) and pacsin2 (syndapin II) (Figure 1) [1]. Caveolae are believed to serve as mechano-sensors and regulators of lipid homeostasis and dysfunction is strongly associated with cardiovascular disease, lipodystrophy and muscular dystrophy. Although present in most cell types, caveolae are abundantly detected in fat, muscle and endothelial cells where they are known to constitute up to 50% of the cell surface area. The abundancy of this typical membrane topology, where the caveolae bulb is connected to the plasma membrane via a neck region, suggests that this state is stabilized and essential for the function of caveolae in many cells.

Due to the apparent general morphological similarity to clathrin-coated vesicles (CCVs), studies of caveolae biogenesis, their structural composition and physiological role have been heavily influenced by the vast and expanding understanding of CCV-formation. Yet, in retrospect, there are many characteristic features that separate these two types of membrane invaginations. CCVs are initiated by the binding of the adaptor-protein complex 2 to cargo receptors at the cell surface. In a sequential process involving more than 50 proteins and enrichment of specific lipid species over time, a clathrin-coated membrane invagination is formed [2,3]. This process is driven by a feed-forward mechanism based on multiple weak interactions between central hub-proteins and accessory proteins, and the forming protein coat could at any time be aborted via disassembly given that key components such as cargo receptors are lacking. The final stage of CCV-formation involves enrichment of Bin-amphiphysin-Rvs167 (BAR) domain-containing proteins, transition of lipid species, actin polymerization and assembly of dynamin, which ultimately leads to fission of the CCV from the cell surface. The vesicle is then uncoated and trafficked to the endosomal system for delivery of the cargo, while the coat- and accessory protein can cycle through another round of CCV formation.

**Figure 2.**
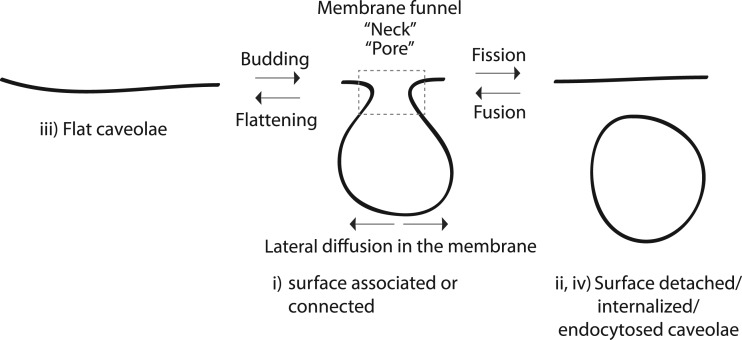
Schematic illustration of caveolae dynamics. Transition in between flat, omega-shaped and scissioned caveolae as well as lateral diffusion of caveolae is depicted by arrows. Dotted square indicates a membrane funnel also referred to as neck, and pore in the literature depending on the research field. i–iv correlates to the states of caveolae as described in the text.

Caveolae on the other hand are formed by the cholesterol-driven oligomerization of caveolins and cavins [4]. The caveolae coat is more stable than the CCV-coat and can remain intact after scission from the cell surface and fusion with the early endosomes. The molecular architecture of the caveolae coat is not fully understood, but recent advances are beginning to unravel the structural composition [5,6]. In addition to the coat components, membrane remodeling proteins such as EHD2 and pacsin2 are stably associated with caveolae [7–9]. Contrary to CCV, caveolae do not seem to be enriched in specific transmembrane receptors, which rather appear to be excluded from caveolae domains in comparison with glycosyl phosphatidylinositol (*GPI*)-*anchored* proteins [10]. Instead, caveolae are heavily enriched in lipids such as cholesterol and glycosphingolipids. The dynamic behavior of caveolae also differ from CCVs and has been described to range from (i) stable surface associated Ω-shaped structures to (ii) short-range cycles of fission and fusion with the plasma membrane, (iii) flattened caveolae and (iv) internalization into early endosomes as well as recycling back to the cell surface [11–15] (Figure 2). In addition, the mobility of both Ω-shaped and flattened caveolae at the cell surface display different levels of lateral diffusion. In this review, we will focus on the transition between (i) and (ii) and the protein and lipid components that facilitate control of the balance between these two states.

The ratio between surface-associated and -dissociated caveolae has been addressed in cultured cells using electron microscopy or total internal reflection fluorescence (TIRF) live cell imaging of fluorescently labeled caveolin [13,16,17]. These methodologies in combination with exogenous labels to track internalization [8,16] and photobleaching experiments to determine mobility [9,10] have been instrumental to our current understanding of caveolae dynamics. Depending on the cell system and the methodology used, ∼1–10% of caveolae have been estimated to be surface released and mobile [10,13,16,18]. Even in such simplified systems, it is difficult to precisely determine the ratio due to the difficulties in tracking single caveolae in time and space and determine if caveolae are surface connected. Specific exogenous labeling of caveolae is currently not possible, and pH-sensitive markers that could differentiate between surface exposed and internalized caveolae do not work due to the fact that endocytosed caveolae are not acidified. The ratio between surface associated and released caveolae in tissues and organs is even harder to address and the physiological role of these different states of caveolae *in vivo* remains to be elucidated.

Surface connected caveolae are characterized by a 20–50 nm thick region with saddle-like membrane curvature bridging the relatively flat plasma membrane with the highly curved caveolae bulb [19]. Both the fusion and fission of all membrane vesicles in cells goes through such a state, where the donor/acceptor membrane and the vesicular membrane are connected by a narrow membrane funnel *(usually referred to as neck, fusion pore,* Ω*-profile)* (Figure 2). Classically, the membrane funnel has been seen as an intermediate and transient state. The membrane funnel can further open up allowing ‘full fusion' or shrink to drive scission of the vesicle. However, little is known about the molecular mechanisms that stabilize membrane funnels such as surface connected caveolae. Interestingly, specific proteins such as EHD2 [8,20], pacsin2 [21,22] and dynamin2 [23,24] and the lipid phosphatidylinositol 4,5-bisphosphate (PI(4,5)P_2_) [25] have been shown to be enriched at the membrane funnel of caveolae, suggesting that this region constitutes a distinct subdomain connected to the caveolae bulb (Figure 1).

### The role of the caveolae coat in surface stability of caveolae

CAV1 (and CAV3 in muscle cells) and cavin1 are essential for the formation of caveolae, and believed to constitute the minimal caveolae coat machinery required to bend the membrane into invaginated caveolae bulbs [26–29]. As such a membrane curvature generator, the caveolae coat could influence the surface stability of caveolae. However, the mechanism for how they bend the membrane into the typical caveolae bulb-shape is not yet understood. CAV1 has, due to its specific hairpin-like membrane topology, been proposed to induce membrane curvature, which in turn could affect the cell surface stability of caveolae. The expression of CAV1 in *E. coli* was reported to induce the formation of small intracellular membrane vesicles containing caveolin in the bacteria, so-called heterologous caveolae (h-caveolae) [30]. Similarly, cell-free expression of CAV1 induced budding of caveolin containing vesicles [31]. Additional evidence for cavin1-independent caveolin curvature comes from super-resolution data showing that CAV1 can form hemispherical scaffolds in PC3 cells lacking cavin1 [32]. This suggests that caveolin has an intrinsic ability to vesiculate membranes, yet cavin1 is required to generate caveolae bulbs in mammalian cells [27]. Purified cavin1 can induce tubulation of liposomes *in vitro* [27,33,34], but the mechanism is elusive and it is not understood how cavin1 co-assembles with CAV1.

The homologous proteins CAV2 and cavin2–4 are also part of the caveolae coat at levels that vary in between different tissues [33,35,36]. Cavin2–4 interact with cavin1, which forms defined complexes with either cavin2 or cavin3 [13,34]. The stochiometric ratio of cavin complexes and abundance of cavin2, 3 and 4 in the caveolae coat seem to vary in between different tissues [20,37,38], likely reflecting adaptation of the coat. Cavin2 is involved in shaping and the size determination of the caveolae bulb [33] and has been shown to be essential for forming caveolae in certain endothelia [38]. Interestingly, cavin3 seems to directly influence the surface stability of caveolae. Depletion of cavin3 increases the surface stability of caveolae [13], and decreases their intracellular mobility [36]. Cavin3 is enriched at deeply invaginated caveolae and increased levels of cavin3 induce their release from the plasma membrane [13]. Membrane binding of cavin3 is cholesterol dependent, and mediated via the same region that directly interacts with the cholesterol interacting domain of CAV1 [13]. The interaction between cavin3 and CAV1 is not required for caveolae biogenesis, suggesting that it rather plays a regulatory role. This indicates that incorporation of cavin3 in the caveolae coat and concerted assembly of CAV1, cavin3 and cholesterol at the membrane interphase will influence caveolae surface stability. Together, our current understanding points to a caveolae coat composition that is flexible and adapted to different tissue specific properties, which could involve variation in the level of cell surface attachment.

### Oligomerization of EHD2 stabilizes the caveolae neck and restrains caveolae to the cell surface

The ATPase EHD2 has proven to be a main regulator of caveolae surface stability. EHD2 belongs to the dynamin superfamily of large GTPases, which also include the homologous proteins EHD1, 3, and 4. EHD2 was shown to oligomerize into ring-like assemblies at the lipid interphase causing membrane tubulation and remodeling [39]. EHD2 is specifically localized to most of the caveolae in cells under normal conditions [8]. Furthermore, the tissue and differentiation specific expression of EHD2 correlates with the expression of the caveolae coat components, suggesting that EHD2 is a fundamental component required for caveolae function [8,40,41]. Interestingly, in contrast with CAV1 and cavin1, EHD2 is located at the neck of caveolae [8,20] and not detected on internal caveolae vesicles, implying that it has a specific role, which is restricted to surface connected caveolae [8,9]. Depletion of EHD2 in cell lines has been shown to result in surface release of caveolae, which increase their dynamics and mobility, while overexpression restrains caveolae to the cell surface [8,9,12,13], showing that EHD2 play a key role for surface association of caveolae. In gene edited cells, this role was substituted by EHD1 and EHD4 in absence of EHD2, but concurrent removal of EHD1, 2, and 4 resulted in higher caveolae mobility [42]. Recent data obtained from EHD2 knock out mice showed a major increase in surface detached caveolae also *in vivo*, and characterization of cells obtained from the mice confirmed the increased mobility and surface detachment of caveolae in cells lacking EHD2 [40]. Furthermore, this study revealed abnormal uptake of free fatty acids into fat cells in mice lacking EHD2, which highlights the physiological importance of the balance between surface connected and disconnected caveolae.

Mechanistic understanding of the assembly process of EHD2 has shed light on the process for how EHD2 restrains caveolae to the cell surface. In solution, EHD2 is present in a dimeric closed conformation [39], where the full membrane interaction surface and oligomerization sites are hidden [12,43]. ATP-binding and the presence of a lipid interphase allows for a major structural rearrangement of the protein domains [12,43]. Upon electrostatic interactions with lipid head groups, the N-terminus, which occupies a hydrophobic pocket in the G-domain in the closed conformation [44], will instead insert into the lipid bilayer [12]. The removal of the N-terminus facilitates tilting of the helical domains into an open conformation, where the helical tips of the protein insert into the membrane [44]. In this open and membrane inserted conformation, the KPF loop in EHD2 reorients to occupy the freed hydrophobic pocket in the G-domain and thereby generates the oligomerization surface of EHD2 [43]. This stringently controlled oligomerization of EHD2 in an open, membrane bound state is essential to restrain caveolae to the cell surface [44]. EHD2 has been shown to be released upon the mechanical stretching and subsequent flattening of caveolae [42,45], which is consistent with a role of EHD2 in stabilization the caveolae neck. Furthermore, the homologous protein EHD1 has been proposed to prevent scission of CCVs in neuronal cells [46], suggesting that this could be a general function of EHDs. ATP-mediated oligomerization of EHD1 has been shown to drive local bulging of membrane tubules, which at tubule diameters below 25 nm resulted in membrane scission [47]. Comparative analysis revealed that EHD2 has a slower ATPase activity which was proposed to limit the ability to mediate scission. It is currently not known why the loss of EHD2 leads to scission of caveolae. One possibility is that this enables recruitment of other protein to the membrane funnel which results in scission. Another possibility is that without the restrain of EHD2, there is nothing limiting the budding of the caveolae bulb. It is also still possible that EHD2 could be important for scission given the right curvature as found for EHD1.

### Pacsin2 contributes to both formation and surface stability of caveolae

Pacsin2, a protein that belongs to the BAR domain-containing superfamily has also been shown to localize to caveolae. It is expressed in most cell types and ∼35–50% of the caveolae found in HeLa and MEF cells have been reported positive for pacsin2 [7,22]. The protein consists of a N-terminal F-BAR domain and a C-terminal Src homology 3 (SH3) domain spaced with a flexible linker region. It forms a crescent-shaped homodimer that binds and tubulates negatively charged membranes via positively charged residues in the F-BAR domain [48,49]. Pacsin2 localizes to the neck of caveolae [21,22], where it assists in generating curvature. It has been shown that depletion of the protein using siRNA results in reduced amounts of invaginated caveolae that lack the typical narrow neck [7]. Furthermore, in a mouse model that lack the muscle specific pacsin/syndapin III protein, the number of invaginated caveolae structures were vastly reduced even though the levels of CAV3 and cavin1 at the plasma membrane remained unaffected [50].

It seems as if pacsin2 not only functions in shaping the neck of caveolae but that it also stabilizes it to the plasma membrane. As pacsin2 is depleted, caveolae becomes more dynamic and the duration time at the plasma membrane decreases, especially the pool of long-lived caveolae. Furthermore, PKCα-phosphorylation of a serine residue (S313) in the flexible linker region between the BAR and the SH3 domain has been shown to trigger its release from the plasma membrane [51]. The direct dissociation from the neck of caveolae also results in less stable caveolae at the plasma membrane. Interestingly, the shorter duration times of caveolae at the plasma membrane caused by loss of pacsin2 at the neck can be reversed by EHD2 expression [51]. The EH domain of EHD2 has been shown to bind to pacsin2 [8,9] implying that the proteins may function together to stabilize the neck.

Pacsin also binds to the proline-rich domain of dynamin via its SH3 domain [22,52]. Dynamin is a homodimeric GTPase that self assembles and its role in membrane fission during clathrin mediated endocytosis is well documented [53]. Dynamin has been detected at the neck of caveolae [23,24], although it is not yet clear to which extent. Cellular addition of the dynamin inhibitor Dynasore results in immobile caveolae at the plasma membrane [11]. Furthermore, overexpression of the GTPase deficient dynamin mutant (K44A) results in stable caveolae at the plasma membrane [23]. This suggests that in analogy with its role in clathrin coated vesicle scission, dynamin might play a similar role at caveolae. However, much less is known about the specific temporal recruitment, assembly and function of dynamin at caveolae. As caveolae are stable microdomains at the plasma membrane, the actual role of dynamin and its interactions with the funnel stabilizing proteins EHD2 and pacsin2 has yet to be clarified.

### Filamin A couples surface connected caveolae to the actin cytoskeleton which restricts lateral diffusion

Caveolae have been shown to associate with actin filaments in several different cell lines and to co-align with actin stress fibers [19,29,54]. Studies have revealed a direct binding between the N-terminal of CAV1 and the C-terminal of FilaminA (FLNa) [55–57]. FLNa belongs to a family of proteins that functions to cross-link actin. The N-terminal of FLNa poses the actin binding domains whereas the C-terminal harbors the self-association site, creating a V-shaped tail to tail homodimer. Besides associating with actin and CAV1, FLNa also binds several other proteins, one being PKCα, which also has been reported to associate with caveolae [58]. The link between caveolae and the actin cytoskeleton by FLNa convey stability within the plasma membrane and restricts the lateral movement of caveolae. Depletion of FLNa results in an uncontrolled, non-linear movement of caveolae within the plasma membrane [59]. This suggests that attachment to the actin cytoskeleton might influence cell surface confinement of caveolae.

### Lipids affect dynamics of caveolae at the cell surface

Caveolae constitute ordered membrane domains highly enriched in cholesterol, which is essential for both caveolae biogenesis and integrity. Caveolae biogenesis has further been linked to other membrane lipids such as phosphatidylserine [60], glycosphingolipid [61] and sphingomyelin [61]. The high cholesterol content of the caveolar domain is required for their characteristic shape as treatment with cholesterol-binding agents such as methyl-β-cyclodextrin or nystatin leads to a flattening of these structures [8,29,62]. CAV1 is embedded in the inner leaflet of the plasma membrane and has been shown to interact with cholesterol through its scaffolding domain, allegedly through the formation of a cholesterol-binding in-plane helix within the lipid bilayer [63]. In analogy with that the caveolae coat proteins could influence caveolae stability by promoting membrane curvature generation, these core-lipid components would also heavily influence caveolae dynamics. Indeed, exogenously added cholesterol decreased the number of caveolae associated to the plasma membrane and enhanced their mobility [18,64]. Similarly, cell treatment with BODIPY-LacCer complexed to bovine serum albumin (BSA) reduced the number of cell surface-connected caveolae [64]. In these studies, the rate of endocytosis was monitored, rather than association of lipids with caveolae in the plasma membrane. Therefore, it is difficult to ascertain if the observed effects on caveolae dynamics are due to perturbations of cellular lipid homeostasis or whether a more general physical process governs this behavior. Furthermore, cell treatment with BSA-complexed BODIPY-LacCer prior to analysis creates uncertainty regarding the extent and residence time of these lipids in the plasma membrane and/or caveolae and, therefore, their induced effect. Because the mechanism, by which cholesterol and other lipids influence the surface stability of caveolae remains elusive, novel methodologies are required in order to address their specific roles in caveolae dynamics.

Although it is well established that caveolae are specialized lipid nanodomains, very little is known with regards to the subdistribution of lipids between the caveolae bulb and the funnel region. An alternative explanation for caveolae budding could be based on a specific membrane composition. Lipid composition has been proposed to drive scission by inducing phase separation, suggesting that this might be a potential mechanism by which lipids could influence caveolae stability [65]. Especially the specific ordering of chiral and tilted lipids could facilitate this process. However, there are no experiments to date that quantify to which extent different lipid species are sequestered or how they diffuse in and out of the caveolae in living cells. Studies based on subcellular fractionations and quantitative morphological analyses of the caveolae ultrastructural suggested that the caveolar neck forms a structurally distinct domain from the caveolar bulb [19,66], but neither the identity of the lipid components around the bulb nor those around the neck are fully defined. PI(4,5)P_2_ has been shown to be enriched at caveolae [25,67,68], with a particular high concentration at the neck region of deeply invaginated caveolae rather than the bulb [25]. This may imply that its distinct localization is connected to a more specialized function likely in connecting caveolae to the cell surface. A distinct pool of PI(4,5)P_2_ in caveolae was found to have a restricted lateral mobility and was not readily exchangeable with the rest of the plasma membrane [25]. Treatment of cells with angiotensin II, which stimulates the hydrolysis of PI(4,5)P_2_ by phospholipase C, led to a delayed decrease in PI(4,5)P_2_ in caveolae compared with the bulk membrane, suggesting that PI(4,5)P_2_ may be shielded through its localization within caveolae. The recovery of PI(4,5)P_2_ levels in caveolae was slow as shown using electron microscopic labeling technique [25], suggesting that PI(4,5)P_2_ may not be able to freely diffuse between noncaveolar and caveolar membranes. This is in agreement with that EHD2, which is located at the neck, preferentially binds to PI(4,5)P_2._ However, no effects on caveolae stability were observed when PI(4,5)P_2_ levels were manipulated [60]. The very high radius of curvature at the neck of caveolar membranes may indeed contribute to enrichment of certain lipids. Because the curvature changes along the Ω-profile, lipids with a specific preference for negative (phosphatidylethanolamine, phosphatic acid, or diacylglycerol) or positive (lysophosphatidylcholine or phosphatidylinositol phosphates) curvature may enrich in different subdomains of the neck region and so contribute to its characteristic shape [69,70]. Elucidating the lipid composition of these sub-regions will greatly contribute to understanding how lipids drive and control the stabilization of the caveolae membrane profile.

## Perspectives

- The importance of membrane funnel stabilization and surface association of caveolae is consistent with the proposed roles of caveolae as mechanosensitive membrane buffers, platforms for signaling and regulators of lipid homeostasis. Currently, the mechanisms by which proteins facilitate stabilization of caveolae membrane funnels are beginning to be unraveled, which will aid our understanding of their biological role and diseases linked to caveolae dysfunction.
- It is now recognized that the membrane funnel constitutes a subdomain of caveolae, separate from the bulb, where specific proteins such as EHD2 and pacsin2 are enriched. The assembly of EHD2 stabilizes the membrane funnel in an ATP dependent mechanism, which is key to preventing caveolae scission.
- Yet, further understanding of the complete arsenal of proteins and their temporal recruitment to the funnel region is required in order to understand the atypical behavior of these membrane invaginations. It is currently not clear if proteins at the funnel act as a macromolecular complex or if they function as distinct entities that promote either caveolae scission or stabilization. Although dynamin2 has been proposed to mediate caveolae scission, the mechanism and molecular components required to fission the caveolae membrane funnel is still elusive. Furthermore, our understanding of the assembly of the caveolae coat is not sufficient to understand the forces that mediate budding of the caveolae bulb and whether this will influence the membrane funnel and stability of caveolae. Our current understanding points to that the caveolae coat composition is flexible and adapted to different tissue specific properties, which could involve variation in the level of cell surface restraints. Precise structural understanding of how the caveolins and cavins interact to build up the caveolae coat and bend the membranes is required to shed light on the importance of variations in the stochiometric relation of the caveolae coat. A major future area of research involves the caveolae lipid interphase and how lipid species influence the dynamic behavior of caveolae at the cell surface. It is currently not clear if specific lipids are enriched at the bulb and funnel regions, respectively, and if there is lateral diffusion of lipids over the funnel region. The sequestering and phase separation of lipids might heavily impact the stability of the membrane funnel. In addition, further mechanistic studies are needed to fully understand the interplay between caveolae protein components and different lipid species, which will help to elucidate their co-ordinated ability to maintain the typical Ω-shaped morphology of surface associated caveolae.

## Abbreviations

BAR: Bin-amphiphysin-Rvs167
BSA: bovine serum albumin
Caveolin1-3: CAV1-3 phosphatidylinositol 4,5-bisphosphate, PI(4,5)P2
CCVs: clathrin-coated vesicles
EHD2: EH-domain containing protein 2
FLNa: FilaminA
*GPI*: glycosyl phosphatidylinositol
SH3: Src homology 3
